# Risk factors for the development of chronic pulmonary aspergillosis in patients with nontuberculous mycobacterial lung disease

**DOI:** 10.1371/journal.pone.0188716

**Published:** 2017-11-30

**Authors:** Byung Woo Jhun, Woo Jin Jung, Na Young Hwang, Hye Yun Park, Kyeongman Jeon, Eun-Suk Kang, Won-Jung Koh

**Affiliations:** 1 Division of Pulmonary and Critical Care Medicine, Department of Medicine, Samsung Medical Center, Sungkyunkwan University School of Medicine, Seoul, South Korea; 2 Department of Statistics, Samsung Medical Center, Seoul, South Korea; 3 Department of Laboratory Medicine and Genetics, Samsung Medical Center, Sungkyunkwan University School of Medicine, Seoul, South Korea; National Institute of Infectious Diseases, JAPAN

## Abstract

Nontuberculous mycobacterial lung disease (NTM-LD) is increasingly recognized as an important predisposing condition for the development of chronic pulmonary aspergillosis (CPA), but there are limited data on the risk factors for CPA development in NTM-LD patients. We reviewed the medical records of 566 patients who, at the time of diagnosis of NTM-LD, did not have CPA and who received ≥12 months of treatment for NTM-LD between January 2010 and June 2015. Of these patients, 41 (7.2%) developed CPA (NTM-CPA group), whereas the remaining 525 patients did not develop CPA (NTM group). The median time to the development of CPA was 18.0 months from treatment initiation for NTM-LD. The NTM-CPA group was older and had significantly higher proportions of males, current smokers, and patients with a low body mass index (<18.5 kg/m^2^), when compared to the NTM group. Moreover, the NTM-CPA group was more likely to have a history of tuberculosis and chronic obstructive lung disease and to have used inhaled or systemic steroids. In the NTM-CPA group, more than 40% of patients had *Mycobacterium abscessus* complex (MABC) as the cause of NTM-LD, and the fibrocavitary form of NTM-LD was the most common; both associations were higher than in the NTM group. Overall, 17 (3%) patients died, and the NTM-CPA group had a higher mortality rate than did the NTM group (19.5% vs. 1.7%, respectively; *P*<0.001). In a multivariable analysis, old age, male gender, low body mass index, chronic obstructive lung disease, systemic steroids, MABC as the etiologic organism, and the fibrocavitary form of NTM-LD remained significant predictors of development of CPA. In conclusion, CPA occurred in 7.2% of patients after initiation of treatment for NTM-LD, and some risk factors were associated with CPA development. Given the worse prognosis, early diagnosis and treatment of CPA are important in patients with NTM-LD.

## Introduction

Chronic pulmonary aspergillosis (CPA) is a slowly progressing pulmonary infection caused by *Aspergillus* species, usually *Aspergillus fumigatus* [1–5]. CPA typically occurs in middle-aged and elderly individuals with underlying chronic pulmonary diseases, such as previously treated pulmonary tuberculosis [6]. In addition, pulmonary disease caused by nontuberculous mycobacteria (NTM) is increasingly recognized as an important underlying condition associated with CPA [6–11], with some patients having CPA and coexisting NTM lung disease (LD) [11–14]. The treatment of NTM-LD associated with CPA is very difficult, and the prognosis is poor [13].

The development of CPA in patients with NTM-LD poses significant challenges for clinicians because clinical parameters might not accurately differentiate the development of CPA from the aggravation of existing or recurrent NTM-LD after treatment completion, and a misdiagnosis can delay treatment. Moreover, even when the diagnosis of combined CPA and NTM-LD is confirmed, the treatment priority or strategy remains a concern due to the complexity of treatment regimens and potential drug interactions between commonly used antifungal and certain antimycobacterial agents, such as itraconazole and rifamycin, which can compromise the therapeutic effects of the antifungal agents [15,16].

Consequently, it is important to identify predictors of CPA in patients with NTM-LD, in order to improve clinical outcomes. In this study, our goals were to elucidate risk factors for the development of CPA in patients who did not have CPA at the time of treatment initiation for NTM-LD.

## Materials and methods

### Study population

**Fig 1:**
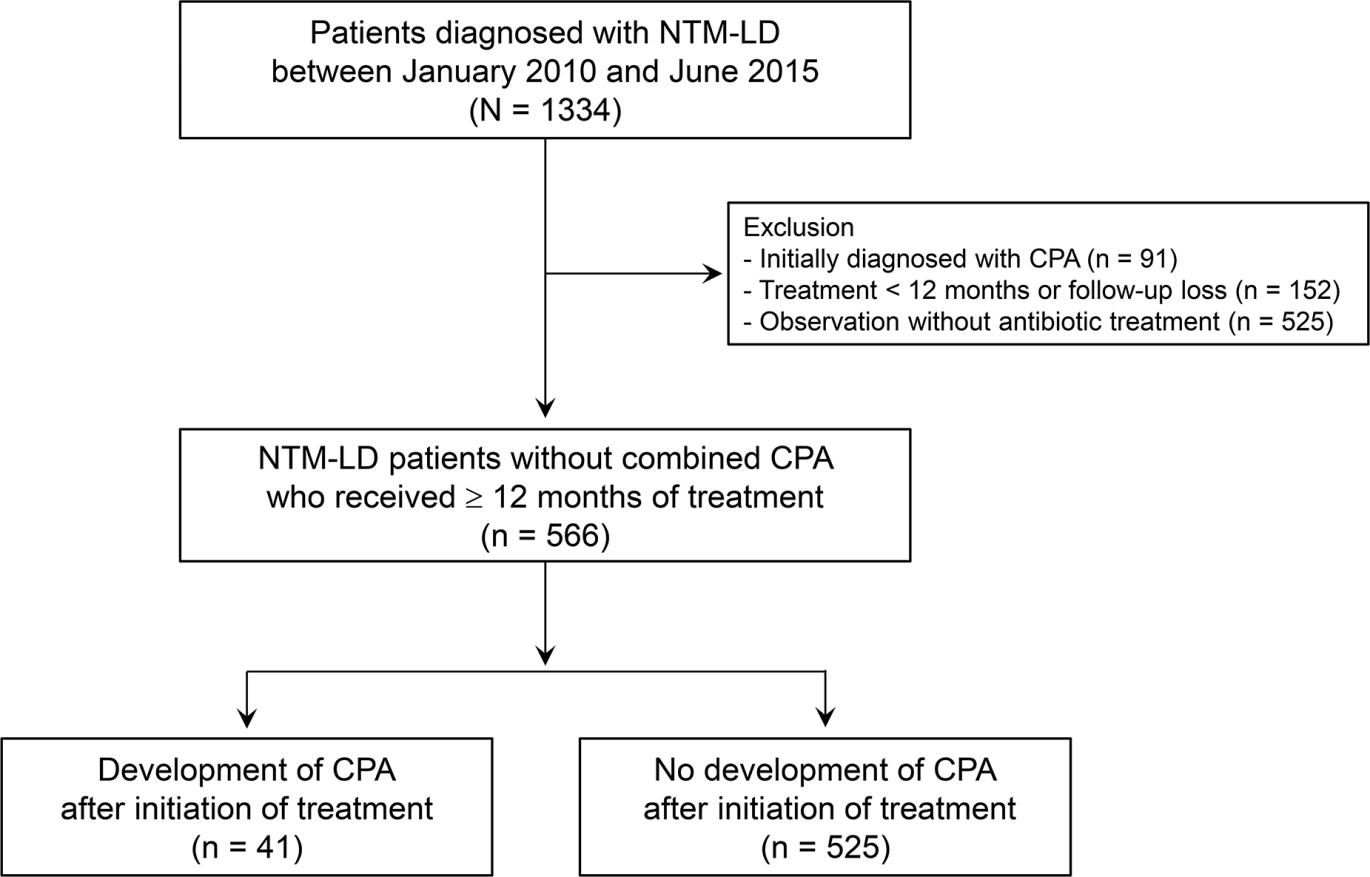
Study patients.

We retrospectively reviewed the medical records of 1,334 consecutive patients diagnosed with NTM-LD from the NTM Registry of Samsung Medical Center (a 1,979-bed referral hospital in Seoul, South Korea) from January 2010 to June 2015. All patients fulfilled the diagnostic criteria of NTM-LD [17]. From this cohort, we excluded patients who were also initially diagnosed with CPA (n = 91), who were treated for fewer than 12 months or lost to follow-up (n = 152), or who did not receive antibiotic therapy (n = 525) (Fig 1). We excluded patients who did not receive antibiotic therapy for NTM-LD because patients who did not need antibiotic treatment for NTM-LD were clinically stable and had a low chance of development of CPA. In addition, patients who were treated for fewer than 12 months were also excluded from this study because these patients were not suitable for assessing the occurrence of CPA, due to the short-term follow-up after initiation of antibiotic treatment for NTM-LD.

Consequently, our study included 566 patients who had NTM-LD without CPA at the time of treatment initiation and who received more than 12 months of treatment for NTM-LD. Of the 566 patients, 254 (44.9%) patients initially had negative results for CPA in serological and microbiological tests, such as the *Aspergillus* precipitin test and/or culture test for *Aspergillus* species. The remaining 312 (55.1%) patients had no radiologic evidence suggestive of CPA, such as cavity or aspergilloma, at the time of initiation of antibiotic treatment for NTM-LD. We divided the study population into two groups: patients who developed CPA (NTM-CPA group) and those who did not develop CPA (NTM group) after treatment initiation during the study period.

This retrospective study was approved by the Institutional Review Board of Samsung Medical Center (IRB No. 2016-11-074). Patient information was anonymized and de-identified prior to analysis, and informed consent was waived. Clinical, radiological, and laboratory data were collected anonymously using standardized forms, with follow-up data last obtained on 27 June, 2016.

### Definitions

Chest radiography and high-resolution computed tomography (HRCT) were available at the time of diagnosis of CPA in all patients. The fibrocavitary form (previously called the upper lobe cavitary form) was defined by the presence of cavitary opacities mainly in the upper lobes. The nodular bronchiectatic form was defined by the presence of bronchiectasis and multiple nodules on chest HRCT, regardless of the presence of small cavities in the lungs [18–21].

The diagnosis of CPA was confirmed when the following findings were present: (i) compatible clinical symptoms, (ii) compatible radiological findings, and (iii) a positive serum *Aspergillus* precipitin test (*Aspergillus fumigatus* IgG ELISA kit; IBL International, Hamburg, Germany) or isolation of an *Aspergillus* species from respiratory samples (i.e., sputum, transtracheal aspirate, or bronchial aspiration fluid) [4,5]. The diagnostic tests for CPA were usually performed when clinico-radiological progression was suspected during antibiotic treatment or after treatment completion of NTM-LD. All of the 566 study patients underwent diagnostic tests for CPA at least once during the study period. Use of steroids was defined as a daily dose of more than 10 mg or a cumulative dose of more than 700 mg of oral prednisolone or a long-term inhaled steroid [22,23].

### Patient management and data collection

The common etiologic organisms of NTM-LD in South Korea are *Mycobacterium avium* complex (MAC, including *M*. *avium* and *M*. *intracellulare*) and *Mycobacterium abscessus* complex (MABC, including *M*. *abscessus* and *M*. *massiliense*) [24–27]. NTM species were identified using polymerase chain reaction (PCR)-restriction fragment length polymorphism analysis of the *rpoB* gene or reverse-blot hybridization of *rpoB* [19,20]. Patients with MAC-LD were mainly treated using a three-drug macrolide-based therapy comprised of macrolides, rifampin, and ethambutol [28–30]. Patients with MABC-LD were mainly treated with two parenteral drugs, such as amikacin plus cefoxitin or imipenem, in combination with oral macrolides [28–30]. Patients diagnosed with CPA were mainly treated with oral itraconazole as the initial agent [11].

### Statistical analysis

All data are presented as median [interquartile range (IQR)] for continuous variables and as number (percentage) for categorical variables. The data were compared using the t-test for continuous variables such as age, and the χ2 test for categorical variables such as sex, body mass index, smoking history, underlying pulmonary disease, use of steroids, etiologic organism, and radiological type. When any cells had an expected count of less than 5, Fisher’s exact test was used instead of the χ2 test. To compare the survival of the NTM-CPA group and the NTM-group, a Kaplan-Meier analysis was performed, and the survival curves were compared by log-rank testing. To assess the risk factors for the development of CPA in patients with NTM-LD, univariate and multivariable analyses with a Cox regression model were performed using those variables that had a *P*-value of <0.05 on univariate analysis in the regression model. Statistical analyses were conducted using PASW software (IBM SPSS Statistics ver. 22; Chicago, IL, USA).

## Results

### Study patients

**Fig 2:**
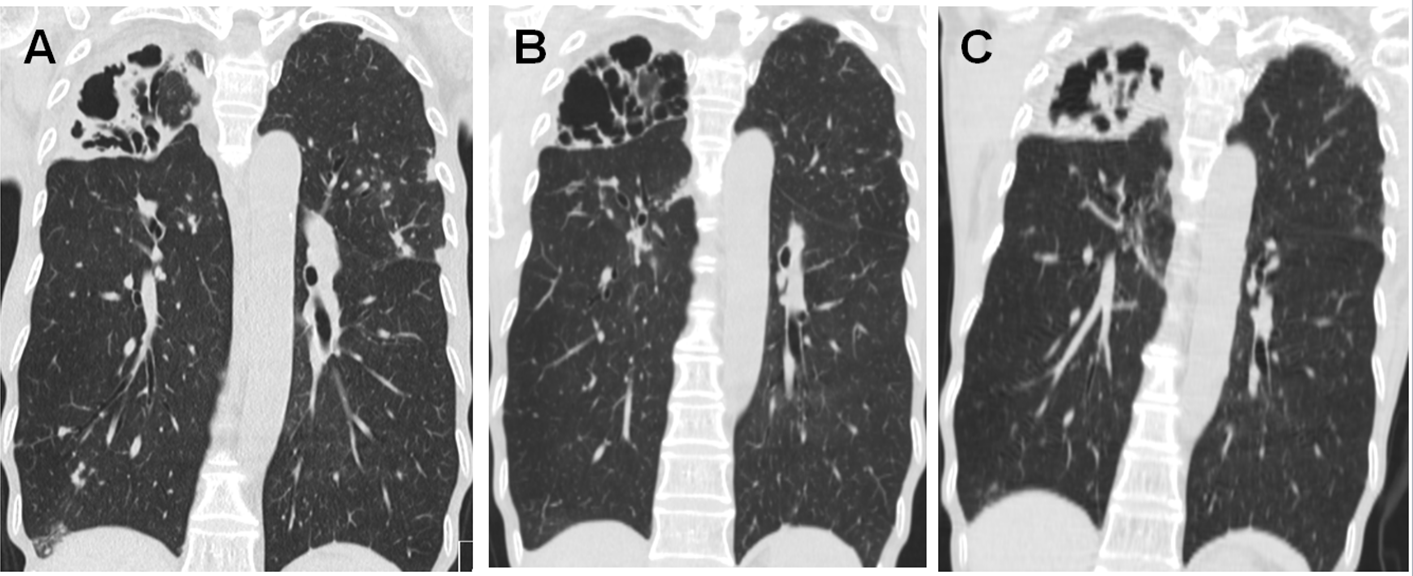
Development of chronic pulmonary aspergillosis in a patient with NTM-LD. Chest computed tomography images are shown for a 69-year-old female patient with *Mycobacterium intracellulare* lung disease. (A) At the time of initiation of antibiotic therapy for *M*. *intracellulare* lung disease, there was a large cavitary consolidation in the right upper lobe. Serum *Aspergillus* precipitin antibody was negative. (B) After 12 months of antibiotic therapy for *M*. *intracellulare* lung disease, cavitary consolidation in the right upper lobe was improved, and sputum cultures for NTM were negative. (C) After 18 months of antibiotic therapy for *M*. *intracellulare* lung disease, the patient’s respiratory symptoms and the consolidation in the right upper lobe were aggravated. Sputum cultures for NTM were negative. However, serum *Aspergillus* precipitin antibody was strongly positive, and chronic pulmonary aspergillosis was diagnosed.

In total, 566 patients who had NTM-LD without combined CPA at the time of treatment initiation and who received more than 12 months of treatment for NTM-LD were identified. Of these, CPA developed in 41 (7.2%) patients after treatment initiation of NTM-LD, with a median follow-up period of 26.0 months (IQR, 18.0–35.0 months) (Fig 2).

**Fig 3:**
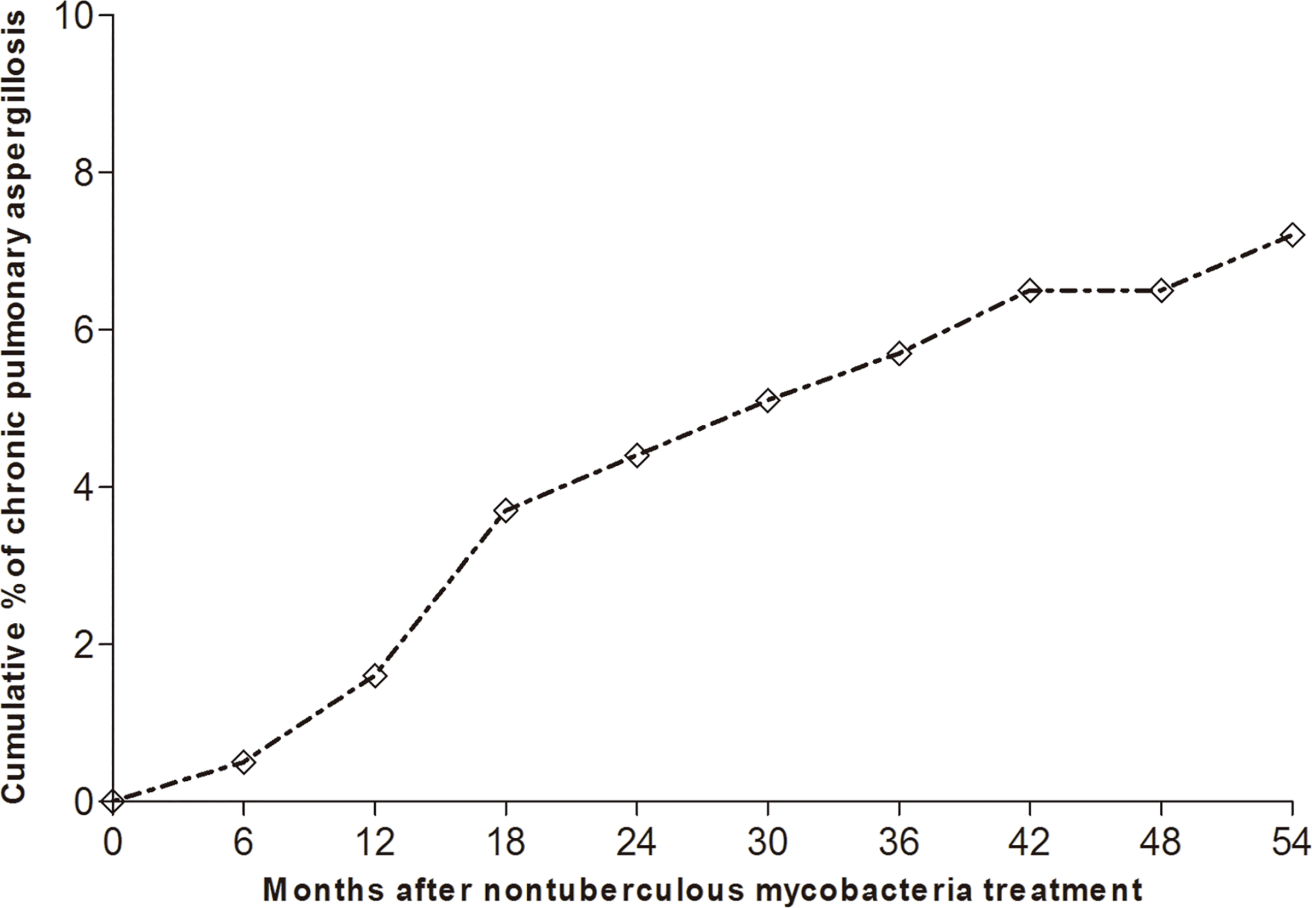
Cumulative percentage of patients who developed chronic pulmonary aspergillosis after initiating treatment for NTM-LD.

All 41 patients who developed CPA had a positive serum *Aspergillus* precipitin test, and nine had positive cultures for *Aspergillus* species, with *A*. *fumigatus* (n = 6) being the most common species and *A*. *flavus*, *A*. *niger*, and *A*. *terreus* found in one patient each. The median time to the development of CPA was 18.0 months (IQR, 12.3–32.0 months) from initiation of treatment for NTM-LD (Fig 3.). Of the 41 patients who developed CPA, 38 (92.7%) were treated with oral itraconazole, two (4.9%) were treated with oral voriconazole, and one (2.4%) did not receive antifungal agents due to an adverse drug reaction.

### Comparison of clinical patient characteristics between the NTM-CPA and NTM groups

We compared the clinical characteristics of the NTM-LD patients who developed CPA to those of NTM-LD patients who did not develop CPA (Table 1). The patients in the NTM-CPA group were more likely to be older (*P* = 0.025), and the proportions of patients with a low body mass index (<18.5 kg/m^2^) and of males were significantly higher in the NTM-CPA group compared with the NTM group (*P*<0.001 for both characteristics). The NTM-CPA group had higher proportions of ex-smokers or current smokers (*P* = 0.035) and more underlying pulmonary disease, such as a history of tuberculosis (*P* = 0.002) or chronic obstructive lung disease (*P*<0.001). Furthermore, compared with NTM patients, NTM-CPA patients used more inhaled steroids (*P* = 0.030) or systemic steroids (*P*<0.001).

**Table 1:** Baseline characteristics of study patients.

Characteristics	Total (N = 566)	NTM-CPA group (n = 41)	NTM group (n = 525)	*P*-value
Age (years)	58 (52–68)	62 (57–71)	58 (51–67)	0.025
Sex (male)	206 (36.4)	27 (65.9)	179 (34.1)	<0.001
Body mass index (<18.5 kg/m^2^)	132 (23.3)	21 (51.2)	111 (21.1)	<0.001
Smoking				0.035
Never smoker	406 (71.7)	24 (58.6)	382 (72.8)	
Ex-smoker	147 (26.0)	14 (34.1)	133 (25.3)	
Current smoker	13 (2.3)	3 (7.3)	10 (1.9)	
Underlying pulmonary disease				
Previous tuberculosis	243 (42.9)	27 (65.9)	216 (41.1)	0.002
Chronic obstructive lung disease	33 (5.8)	13 (31.7)	20 (3.8)	<0.001
Idiopathic pulmonary fibrosis	17 (3.0)	3 (7.3)	14 (2.7)	0.093
Lung cancer	16 (2.8)	1 (2.4)	15 (2.9)	0.876
Underlying extrapulmonary disease				
Diabetes mellitus	28 (4.9)	2 (4.9)	26 (5.0)	0.983
Chronic liver disease	29 (5.1)	4 (9.8)	25 (4.8)	0.150
Chronic heart disease	31 (5.5)	1 (2.4)	30 (5.7)	0.718
Chronic kidney disease	10 (1.8)	1 (2.4)	9 (1.7)	0.532
Rheumatic disease	17 (3.0)	1 (2.4)	16 (3.0)	0.999
Use of inhaled steroids	10 (1.8)	3 (7.3)	7 (1.3)	0.030
Use of systemic steroids	10 (1.8)	5 (12.2)	5 (1.0)	<0.001
Etiologic organism of NTM-LD				0.002
*M*. *avium* complex	373 (65.9)	19 (46.3)	354 (67.4)	
*M*. *avium*	187/373	8/19	179/354	
*M*. *intracellulare*	186/373	11/19	175/354	
*M*. *abscessus* complex	114 (20.1)	17 (41.5)	97 (18.5)	
*M*. *abscessus*	45/114	9/17	36/97	
*M*. *massiliense*	69/114	8/17	61/97	
Others	79 (14.0)	5 (12.2)	74 (14.1)	
Radiological type of NTM-LD				<0.001
Nodular bronchiectatic form	435 (76.8)	16 (39.0)	419 (79.8)	
Fibrocavitary form	105 (18.6)	24 (58.5)	81 (15.4)	
Unclassified form	26 (4.6)	1 (2.5)	25 (4.8)	

Data are shown as median (interquartile range) or number (%).

CPA, chronic pulmonary aspergillosis; NTM, nontuberculous mycobacteria; LD, lung disease.

The etiologic organisms of NTM-LD differed between the NTM-CPA and NTM groups. In the NTM-CPA group, more than 40% of patients (17/41) had MABC as the cause of NTM-LD, which was a significantly higher proportion compared to the NTM group (41.5% vs. 18.5%, respectively; *P* = 0.002). Radiologically, the fibrocavitary form was significantly more common in the NTM-CPA group compared with the NTM group (58.5% vs. 15.4%, respectively; *P*<0.001).

### Comparison of treatment outcomes of patients in the NTM-CPA and NTM groups

**Fig 4:**
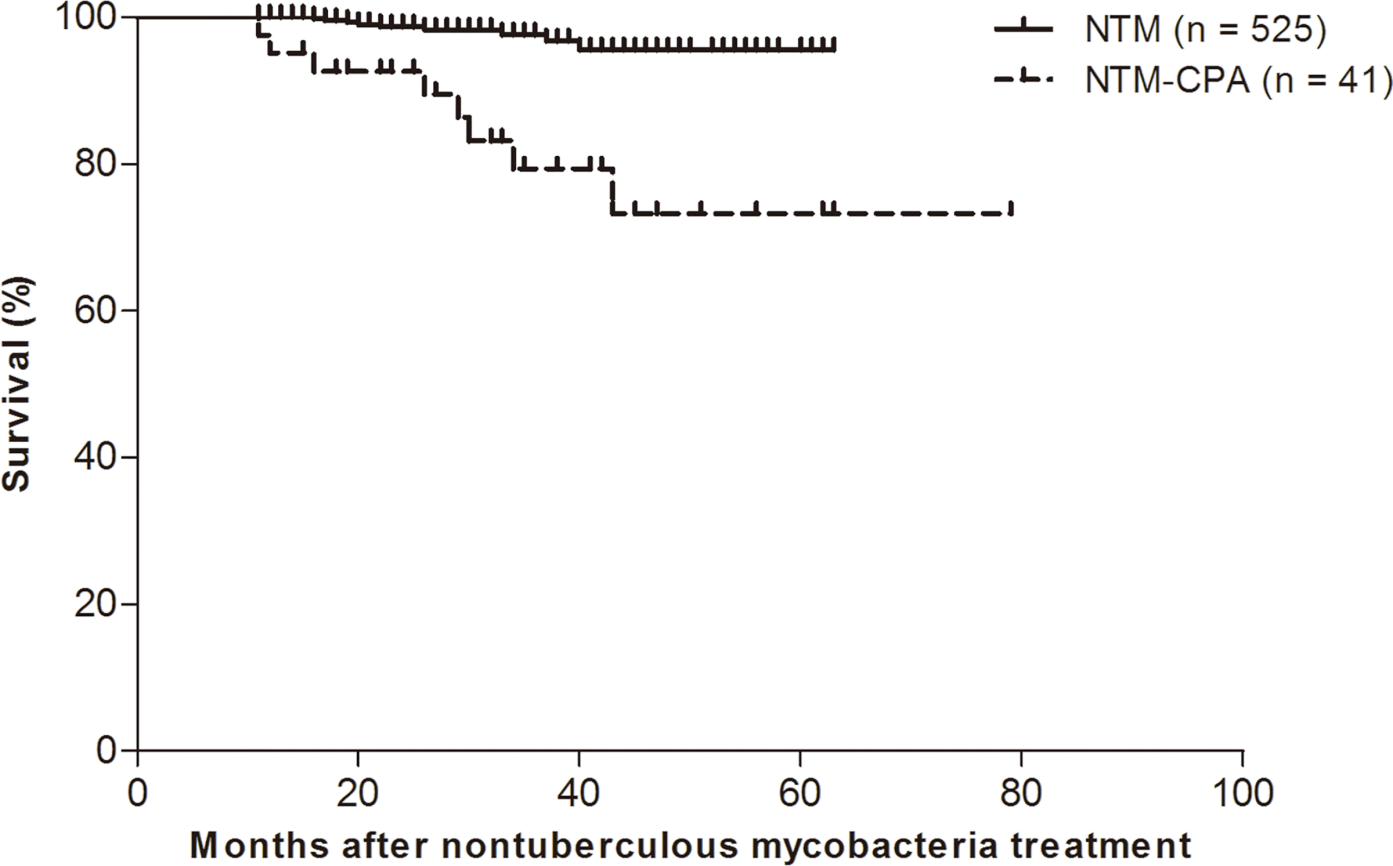
Survival curves in study patient groups using Kaplan-Meier analysis.

In total, 17 (3%) of the study patients died during a median follow-up period of 26.0 months (IQR, 18.0–35.0 months). The NTM-CPA group had a higher mortality rate than the NTM group [8/41 (19.5%) vs. 9/525 (1.7%), respectively; *P*<0.001], and the survival curves of the NTM-CPA and NTM groups generated using Kaplan–Meier analysis differed significantly (log-rank test, *P*<0.001; Fig 4).

### Evaluation of risk factors for development of CPA after NTM-LD treatment

To evaluate the risk factors for development of CPA after NTM-LD treatment, univariate and multivariate analyses with a Cox regression model were performed (Table 2). On univariate analysis, old age (≥60 years), male gender, low body mass index (<18.5 kg/m^2^), being an ex-smoker, previous history of pulmonary tuberculosis, chronic obstructive lung disease, use of systemic steroids, MABC as the etiologic organism, and the fibrocavitary form of NTM-LD were significantly associated with development of CPA. On multivariable analysis, old age (≥60 years, *P* = 0.033), male gender (*P* = 0.006), low body mass index (<18.5 kg/m^2^, *P*<0.001), chronic obstructive lung disease (*P*<0.001), use of systemic steroids (*P*<0.001), MABC as the etiologic organism (*P* = 0.004 for *M*. *abscessus* and *P* = 0.001 for *M*. *massiliense*), and fibrocavitary form (*P*<0.001) were associated with development of CPA after NTM-LD treatment.

**Table 2:** Univariate and multivariable analyses of risk factors for development of CPA.

Variable	Univariate HR (95% CI)	*P*-value	Multivariable Adjusted HR (95% CI)	*P*-value
Age (≥60 years)	2.373 (1.255–4.488)	0.008	2.433 (1.074–5.512)	0.033
Male	3.888 (2.030–7.445)	<0.001	3.760 (1.456–9.711)	0.006
Body mass index (<18.5 kg/m^2^)	3.533 (1.914–6.522)	<0.001	4.965 (2.452–10.055)	<0.001
Smoking				
Never smoker	reference		reference	
Ex-smoker	1.948 (1.004–3.780)	0.049	0.607 (0.253–1.456)	0.263
Current smoker	2.694 (0.805–9.019)	0.108	0.183 (0.041–0.820)	0.050
Previous tuberculosis	2.710 (1.417–5.184)	0.003	0.787 (0.355–1.745)	0.566
Chronic obstructive lung disease	8.783 (4.526–17.045)	<0.001	7.004 (3.157–15.537)	<0.001
Use of systemic steroids	8.978 (3.486–23.118)	<0.001	15.000 (4.743–47.439)	<0.001
Etiologic organism of NTM				
*M*. *avium*	reference		reference	
*M*. *intracellulare*	1.436 (0.577–3.571)	0.437	0.429 (0.157–1.177)	0.100
*M*. *abscessus*	2.936 (1.099–7.843)	0.032	5.123 (1.663–15.776)	0.004
*M*. *massiliense*	4.125 (1.586–10.730)	0.004	5.527 (1.937–15.776)	0.001
Others	1.495 (0.489–4.573)	0.480	0.901 (0.268–3.024)	0.866
Radiological type of NTM				
Nodular bronchiectatic form	reference		reference	
Fibrocavitary form	5.971 (3.168–11.254)	<0.001	7.932 (3.242–19.407)	<0.001
Unclassified form	1.313 (0.173–9.945)	0.792	0.631 (0.064–6.198)	0.692

CPA, chronic pulmonary aspergillosis; NTM, nontuberculous mycobacteria; HR, hazard ratio; CI, confidence interval.

## Discussion

One of the most notable findings in our study was that certain clinico-microbiological factors, such as old age, male gender, low body mass index, chronic obstructive lung disease, use of systemic steroids, MABC as the etiologic organism, and fibrocavitary form, were associated with development of CPA in patients with NTM-LD. This constitutes important information that can aid in the early diagnosis of CPA, which can develop as a complication in patients with NTM-LD.

CPA is a chronic, progressive infection caused by *Aspergillus* species, which are ubiquitous fungi. Numerous underlying conditions that weaken the immune system are associated with CPA development, and studies have consistently reported that preexisting pulmonary disease is an important underlying condition, with mycobacterial pulmonary infection being one of the most common conditions associated with CPA occurrence [4–6]. In our previous study that evaluated the underlying lung disease status of 70 CPA patients, we found that 45.7% of CPA patients had previous or concurrent NTM lung disease [11]. In addition to investigating the etiological aspects of NTM-LD and CPA, awareness of risk factors for CPA could be useful for deciding the treatment strategy in patients with NTM-LD, especially given that CPA has a poor prognosis. In fact, several studies have described a very poor prognosis for CPA. Ohba *et al*. described 21 (50.0%) deaths among 42 CPA patients with a mean follow-up of 28.7 months [10], and Nam *et al*. reported a median survival of only 62 months in 43 CPA patients [31]. These data suggest the importance of early prediction of CPA co-infection when treating NTM-LD.

To date, however, there are only limited data on the risk factors for developing CPA in patients with NTM-LD. Recently, Takeda *et al*. reported that nine of 82 (11.0%) patients with NTM-LD developed CPA during their observation period, and cavity formation and steroid therapy were associated with CPA occurrence [13]. However, their study differed from ours in that they did not include the serum *Aspergillus* precipitin test among the diagnostic criteria for CPA and instead used only mycological examination, such as positive isolation of *Aspergillus* species by culture or pathological examination. Given that the *Aspergillus* IgG antibody test is the most accurate microbiological test available, as described in recent guidelines [4,5], some of the nine NTM-LD patients with CPA in the previous study [13] might only have been colonized with *Aspergillus*. Moreover, four of the nine patients in the study by Takeda *et al*. were simultaneously diagnosed with both CPA and NTM-LD, whereas our study included only patients who developed CPA during the observational period after NTM-LD diagnosis. Finally, their analysis included a relatively small number of CPA patients. Kobashi *et al*. also reported nine cases of CPA occurring during the follow-up for MAC lung disease, seven of whom had poor clinical outcomes [8]; however, that study did not evaluate the predictors of CPA occurrence. Therefore, we believe that our data have clinical importance for the management of NTM-LD patients who have potential risk factors for development of CPA.

Recent studies of CPA have reported that obstructive lung disease, lung cavities, and steroid use are common underlying conditions in patients who develop this infection. Interestingly, we found that, compared with MAC-LD, MABC-LD was more often associated with development of CPA. In a study of 139 cystic fibrosis patients, MABC infection showed a greater tendency than MAC infection to be associated with allergic bronchopulmonary aspergillosis [32]: five (3.6%) patients met the criteria for MABC-LD, and all five had allergic bronchopulmonary aspergillosis. However, there is insufficient understanding of the microbiological association between MABC infection and CPA occurrence. Given that MABC is highly resistant to various antibiotics compared with MAC, and infections caused by MABC are still considered to be chronic incurable diseases, our findings may be associated with the persistent lung damage caused by MABC in patients with these infections; further studies are needed to clarify this potential confounding factor.

To date, however, despite the importance of identifying aspergillosis, there are still limited data on the incidence of CPA in NTM-LD patients, which hampers estimation of the optimal screening interval for CPA in these patients. Takeda *et al*. reported that 11.0% of 82 NTM-LD patients developed CPA during the observation period in their recent study [13], and Kobashi *et al*. found that the mean time to diagnosis of CPA from the diagnosis of MAC lung disease was 36.0 months in nine cases [8]. In our study, 7.8% of patients who initiated antibiotic therapy for NTM-LD developed CPA, and the median time to development of CPA was 18.0 months (IQR: 12.3–32.0 months) after initiation of treatment for NTM-LD. Based on our data, we cautiously suggest that, in NTM-LD patients with any of the identified risk factors, screening more often than every 12 months using the serum *Aspergillus* precipitin test or cultures might help to facilitate the early diagnosis of CPA, although further studies are needed.

Our study had several limitations. First, our analysis was retrospective and confined to a single center. Second, not all patients underwent regular screening tests for CPA, mainly due to a lack of guidelines and knowledge of risk factors associated with CPA, although the majority of patients, especially those with cavitary NTM-LD, were tested at least once during the follow-up period to assess for *Aspergillus* infection using the serum *Aspergillus* precipitin test or culture. Therefore, some CPA cases might have been missed. Third, there was a possibility that the patients who had poor treatment responses for NTM-LD, especially MABC-LD, might more frequently undergo CPA testing compared with patients with MAC-LD. Fourth, MABC-LD can develop during or after treatment for MAC-LD, or vice versa [20,33], and, in our study, the etiologic organisms were determined at the time of initiation of antibiotic therapy for NTM-LD. Therefore, specific etiologic organisms may have been incorrectly associated with the development of CPA in some patients. Lastly, considering the chronic nature of CPA and NTM-LD, the follow-up duration might have been relatively short.

## Conclusion

In conclusion, in our study, 7.2% of NTM-LD patients developed CPA after initiation of antibiotic therapy for NTM-LD. Clinico-microbiological factors associated with development of CPA in patients receiving therapy for NTM-LD were old age, male gender, low body mass index, chronic obstructive lung disease, systemic steroid use, MABC as the etiologic organism, and the fibrocavitary form of NTM-LD. These data constitute important information for the early detection of CPA in the context of NTM-LD, which might help improve treatment strategies.

## Abbreviations

CPA: chronic pulmonary aspergillosis
IQR: interquartile range
LD: lung disease
MABC: *Mycobacterium abscessus* complex
MAC: *Mycobacterium avium* complex
NTM: nontuberculous mycobacteria
